# A Study of Personal Health Record User’s Behavioral Model Based on the PMT and UTAUT Integrative Perspective

**DOI:** 10.3390/ijerph14010008

**Published:** 2016-12-23

**Authors:** Hui-Lung Hsieh, Yu-Ming Kuo, Shiang-Ru Wang, Bi-Kun Chuang, Chung-Hung Tsai

**Affiliations:** 1 Chu Shang Show Chwan Hospital, Nantou 557, Taiwan; cs6020@csshow.org.tw (H.-L.H.); cs7500@csshow.org.tw (B.-K.C.); 2 Department of Marketing and Distribution, Tzu Chi University of Science and Technology, Hualien 970, Taiwan; ss248@ems.tcust.edu.tw; 3 Department of Business Administraiton, National Dong Hwa University, Hualien 974, Taiwan; shiang2216@gmail.com; 4 Department of Health Administration, Tzu Chi University of Science and Technology, Hualien 974, Taiwan

**Keywords:** personal health record, protection motivation theory, UTAUT model

## Abstract

The personal health record (PHR) is a system that enables borderless medical care services by combining technological innovation and human consideration. This study explored factors affecting the adoption of PHR from technical, medical, and social perspectives according to the Protection Motivation Theory (PMT) and Unified Theory of Acceptance and Use of Technology (UTAUT) model. A survey using a structured questionnaire was subsequently conducted, which produced the following results: (1) The PMT and UTAUT were effective at predicting PHR usage behaviors; (2) Perceived ease-of-use was the most decisive factor influencing the use of PHR, followed by self-efficacy and perceived usefulness; and (3) Behavioral intention for PHR was significantly and positively correlated with usage behavior. From the obtained results, this study recommends that health authorities and medical institutions promote self-efficacy in the use of PHR to improve the levels of behavioral intention and usage behavior among the people. Additionally, medical care institutions are recommended to promote health management and preventive healthcare concepts to help improve public acceptance of the PHR system as a means to self-manage their health. Finally, community centers, medical institutions, and health authorities are urged to work together to enhance public medical knowledge and pool resources for the PHR system, both of which are essential for improving the popularity of the PHR, public quality of life, and the effectiveness of health management.

## 1. Introduction

Taiwan has become an aging society since 1994, and in 2014, senior citizens made up 12% of its entire population. Although the aging index in Taiwan is still lower than those in Canada, Japan, and European countries, it is already higher than those in the United States, Australia, New Zealand, and most other Asian countries. Currently, people are paying more attention to their own health conditions with the advance in technologies and standard of living, for which numerous mobile applications (a cloud personal health monitoring systems) have been released. Among these mobile applications is the personal health record (PHR) system, whose design is based on the concept of preventive healthcare and is the most noteworthy of all [1].

The American Health Information Management Association has defined the PHR as “An electronic system, generally available lifelong health information resource, which can help people to make a healthy decision”. People use the information by PHR, which comes from health care providers and individuals. Another popular definition, from the Healthcare Information and Management Systems Society, describes it as “a universally accessible, layperson comprehensible, lifelong tool for managing relevant health information, promoting health maintenance and assisting with chronic disease management” [2].

A PHR enhances a patient’s awareness of personal governance in health management, and reduces the wastage of medical resources that occurs from unnecessarily repeating tests and prescriptions. Furthermore, the adoption of the PHR system can help resolve the problem of unequal medical resource distribution. For example, residents in remote areas that lack medical resources (e.g., facilities and manpower) are compelled to seek medical attention from various places, resulting in a wide dispersal of personal medical records that are not readily available for physicians to review a patient’s medical history and medication record. Therefore, transmitting medical data into an online PHR is instrumental for physicians and health care providers to effectively communicate with patients, and improves the physician-patient relationship as well as patient satisfaction [3,4,5]. PHR provides an integrated and broad range of health-related information, including disease symptoms, medical problems, allergies, vaccination history, medical records and medical provider recommendations.

However, the promotion of the PHR system faces considerable difficulties in the current medical practice framework, and only a small amount of data were recorded in its initial stages [6]. Such difficulties can be gradually resolved through the long-tern accumulation of data and platform integration, but user acceptance is a critical issue that must be addressed first. Presently, acceptance of the PHR system is low, even in the United States, despite many private enterprises offering this innovation for free to the general public. Some studies have indicated that the PHR system must be incorporated into existing medical and social infrastructures for it to gain patient acceptance [7]. In other words, both medical and social factors have to be considered, rather than focusing merely on technical factors, to improve understanding of the key factors impeding the adoption of the PHR system.

Among the numerous theories on health behaviors, the Protection Motivation Theory (PMT) has seen widespread use in this field. We used PMT as the main theory to predict behavioral intention. In order to emphasize the context of PHR, we needed to use Unified Theory of Acceptance and Use of Technology (UTAUT) to explain behavioral intention. Because the Technology Acceptance Model (TAM) is chiefly used to explain usage behaviors regarding information systems or health-related technologies [8,9], it has further been expanded into the Unified Theory of Acceptance and Use of Technology (UTAUT) by adding social influence as a variable, while retaining the original variables in the TAM [10], which makes it more comprehensible and encompassing. Accordingly, this study adopted the PMT and UTAUT models to explore the PHR usage behaviors from technical, medical, and social perspectives, with the goal of identifying the decisive factors that could facilitate improved public health and welfare through a widely accepted PHR system.

### 1.1. Protection Motivation Theory

The PMT is often used as the theoretical basis for the study of personal protective behaviors. It was first proposed by Rogers [11], who reasoned that an individual facing a treatment event would undergo a threat appraisal process and a coping appraisal process for self-protection. The theory consists of five core variables, namely perceived severity, perceived vulnerability, response efficacy, self-efficacy, and response costs. In a later study, Rogers [12] advanced that an individual’s intention of performing health behaviors would be determined by his or her appraisal of perceived severity and vulnerability (i.e., threat appraisal), and his or her appraisal of the efficacy of coping with the threat of certain health behaviors (i.e., coping appraisal). Therefore, the PMT is a social cognitive model that prioritizes relevant perceptions to predict the outcome of certain health behaviors.

The PMT is in fact an extension and expansion of the Health Belief Model (HBM). According to the HBM, a behavior intention is determined by the perceived importance of its goal, and the perceived feasibility of reaching this goal through the behavior. When the goal is to avoid a health problem, the potential severity of and susceptibility to this problem must be perceived before an action is taken. Additionally, health must be achievable through the reduction of threat (perceived benefits), with little obstruction (perceived barriers) in the problem-solving process [13]. Both these theories contain appraisals of the benefits and costs of performing alternative behaviors; such similarity indicates that the PMT retains the four cognitive factors (i.e., severity, susceptibility, perceived benefits, and perceived barriers) of the HBM, but the PMT is capable of a more thorough and in-depth analysis of the mechanisms and process of behavior change. Thus, the present study believed that the intention of health behaviors could be predicted through threat appraisal, coping appraisal, and cognitive variables. Specifically, threat appraisal would be determined by the perceived susceptibility and severity of a certain disease, whereas coping appraisal would be determined by self-efficacy and preventive behavior efficacy. Perceived susceptibility is an individual’s perception of the risk of contracting a certain disease, and perceived severity addresses an individual’s perception on the disease’s impact to daily life. Self-efficacy refers to an individual’s self-confidence in performing a responsive behavior, and response efficacy reflects the individual’s belief in effectively reducing the threat through that responsive behavior. Finally, response costs reveal an individual’s perception of the costs corresponding to the responsive behavior.

Although the PMT has been successfully applied in a wide range of health behavior interpretations and predictions [7,14], its application in studies on PHRs is still rather limited. Therefore, the present study incorporated the antecedent variables of the PMT as factors affecting the acceptance of PHRs.

### 1.2. Unified Theory of Acceptance and Use of Technology

According to Davis [15], the TAM is a theoretical lens that can be used to explain the impact of external factors on behavioral intention. However, Venkatesh [16] determined that many of the variables have their own unique properties that are applicable in a wide range of fields, which resulted in the development of the integrated UTAUT model. The UTAUT model contains three constructs that have been recognized as decisive factors for behavioral intention: performance expectancy, effort expectancy, and social influence. Behavioral intention, along with the facilitating conditions, constitute UTAUT’s two constructs from the TAM that are deemed decisive factors for usage behaviors: perceived ease-of-use (PEOU) and perceived usefulness (PU). PEOU is incorporated into the construct of effort expectancy, and PU into performance expectancy; the subjective norm, a variable that is not included in the TAM, is incorporated into the construct of social influence. In short, PEOU refers to the perceived degree of simplicity regarding the use of a particular information technology, whereas PU refers to the perceived degree of performance enhancement gained by using a particular information technology; subjective norm refers to the degree to which people in key positions consider whether an individual should perform a particular behavior.

The UTAUT model has been successfully applied in studies of various health-related technologies, including information systems [17,18,19,20,21,22,23,24], health facilities [25], and telehealth care services [26,27,28], which indicates that the model is viable for assessing health-related technology usage behaviors. Therefore, the present study sought to determine the perceived benefits of PHR for a user and the factors affecting usage behaviors as references to guide the system’s future improvement.

### 1.3. Interrelationship between Variables and Hypotheses Development

#### Relationship between Self-Efficacy and Perceived Usefulness and Perceived Ease-of-Use

Previous studies on electronic health records have found that the decisive factors that influenced physicians’ acceptance included PU and PEOU. Moreover, the use of computer self-efficacy as an antecedent variable has a significantly positive impact on PEOU, which in turn affects PU. This suggests that if a physician considered him or herself proficient with information technology, operating the electronic health records system would feel easier if additional training were given; furthermore, the physician’s PU of the system would be enhanced [18]. Kowitlawalul, Chan, Pulcini, and Wang [22] also demonstrated that nursing students’ levels of self-efficacy with the electronic health records system would have a significant positive impact on their PU and PEOU, which implies that the more self-efficacy that is gained through training, the more the system is perceived to be easy to operate. This also further increases eagerness to learn (PU).

The above observations clearly indicate that the TAM is applicable to health-related technological studies, and that self-efficacy is significantly correlated with PU and PEOU. The present study hence proposed the following hypotheses:

H1: Self-efficacy positively influences perceived usefulness.

H2: Self-efficacy positively influences perceived ease-of-use.

### 1.4. Relationship between Social Support and Perceived Benefits Relationship between Behavioral Intention and Perceived Usefulness, Perceived Ease-of-Use, and Subjective Norm

Numerous studies have verified that the UTAUT model can be used to assess health behaviors. For example, studies on PHRs have revealed that if patients found recommendations in the PHR system useful, and the system was also easy to operate, usage behaviors increased. In addition, patients have also reported that they asked their physicians more health-related questions after their experiences with the PHR system. Health care providers also hold the system in high regard, because it offers access to patients’ medical records and influences their medical behaviors [24]. A study on electronic health records discovered that although many countries have adopted a PHR system, physicians’ acceptance has posed considerable limitations to its implementation. Four models were subsequently used to explore physicians’ acceptance of the technology, and revealed that decisive factors influencing the physicians’ acceptance included PU, PEOU, and subjective norms. According to the psychosocial model, social and professional norms are the most decisive factor for physicians’ acceptance. If a physician considered the use of the system an adequate medical practice, and at the same time earned the support of peers and patients, the physician’s usage behavior of the system would be increased [18]. Another study obtained similar findings that the higher a physician’s self-efficacy, PU, and subjective norm levels were, the more likely he or she would be to adopt the electronic health records system; the subjective norm, in particular, was found to be the most decisive factor [29]. Furthermore, a study that examined nursing students’ experiences with electronic health records systems, demonstrated that PU and PEOU were positively correlated with attitude, and PU was also positively correlated with behavioral intention [22].

According to the above discussion, TAM can be effectively applied to health-related technology studies, and variables such as PU, PEOU, and subjective norm significantly influence behavioral intention. Accordingly, the present study proposed the following hypotheses:

H3: Perceived usefulness positively influences behavioral intention.

H4: Perceived ease-of-use positively influences behavioral intention.

H5: Subjective norm positively influences behavioral intention.

### 1.5. Relationship between Behavioral Intention and Self-Efficacy, Perceived Severity, Perceived Susceptibility, Response Efficacy, and Response Costs

The perceived severity, perceived susceptibility, self-efficacy, response efficacy, and response costs are variables in the PMT that are effective in predicting short- or long-term health behaviors, and have all been successfully applied in studies on various public health issues. For example, a study found that if people had higher levels of perceived severity, perceived susceptibility, and self-efficacy in their awareness of the health risks of an unhealthy diet, their dietary behaviors would become healthier as a result; self-efficacy was found to the most decisive factor in this regard [30]. A study on salt consumption found that people’s perceived severity of hypertension indirectly affected their salt consumption [31]. In a study on consumer behavior regarding ferric soy sauce, researchers determined that consumers purchased more of these products when their perceived susceptibility and perceived severity of iron-deficient anemia was higher [32]. Another study, on the behavioral intention of consuming iodized foods to prevent mental retardation, demonstrated that higher self-efficacy among parents enhanced the behavioral intention to improve their children’s iodine consumption; by contrast, higher response costs reduced the school administrator’s behavioral intention [33]. In a study on the use of dietary supplements to prevent intellectual deterioration, higher perceived severity, self-efficacy, and response efficacy levels were determined to increase the behavioral intention toward consuming supplements [34]. Finally, a study on food hygiene revealed that people with higher levels of self-efficacy and response efficacy in food preparation increased their behaviors toward preparing food properly, preventing cross infection, cooking at appropriate temperatures, and avoiding unsafe food [35].

The preceding hypothesis has been found equally valid in PHR research. One study found that patients with higher perceived severity, and patients with a strong motivation in regaining health, would be more willing to share their medical histories with the hospital medical staff [36]. Another study determined that when students’ perceptions of system vulnerability (susceptibility) was raised, and when they exhibited higher self-efficacy in using the system, their intention to follow the guidelines was enhanced; conversely, the intention declined when students’ perceived response costs increased [37].

Researchers have clearly demonstrated that the PMT is effective for health-related studies, and that self-efficacy, perceived severity, perceived susceptibility, response efficacy, and response costs are variables that markedly influence behavioral intention. The present study therefore proposed the following hypotheses:

H6: Perceived severity positively influences behavioral intention.

H7: Perceived susceptibility positively influences behavioral intention.

H8: Self-efficacy positively influences behavioral intention.

H9: Response efficacy positively influences behavioral intention.

H10: Response costs negativity influences behavioral intention.

### 1.6. Relationship between Behavioral Intention and Usage Behavior

According to the TAM, attitude is influenced by PU and PEOU, and behavioral intention, which affects the usage behavior performed, is influenced by attitude and PU [15]. For example, research determined that a higher level of physicians’ acceptance and behavioral intention increased their usage of electronic health record systems [29]. Similar findings were noted in a study on adolescents exposed to cigarettes, which found that adolescents with higher self-efficacy to avoid cigarettes exhibited a lower intention to smoke and fewer instances of smoking [38,39]. These examples suggest that behavioral intention has a substantial influence on usage behaviors. Therefore, the present study proposed the following hypothesis:

H11: Behavioral intention positively influences usage behavior.

**Figure 1:**
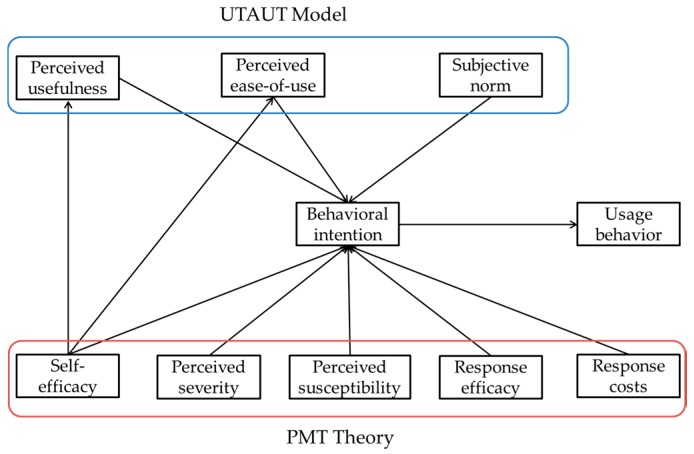
Research model. UTAUT: Theory Unified Theory of Acceptance and Use of Technology; PMT: Protection Motivation (UTAUT).

Based on the above 11 hypotheses, this study presents a complete model (Figure 1).

## 2. Research Method

The online PHR system of a regional hospital in Zhushan, Nantou County was the subject of the present study. The front end of the system is a community telehealth system that gathers and transfers vital sign data of community residents into the hospital’s patient database which, after incorporating with the back-end system (i.e., the hospital’s information system), becomes a webpage-based PHR system accessible through mobile devices (e.g., smartphones and tablets). The system allows its users to review the historical data of their vital signs (pulse rate, temperature, respiration rate, and blood pressure), as well as their medical records, such as the diagnoses, tests, prescriptions, and treatments they have received. This increases the users’ understanding of their health conditions and enables them to monitor the long-term progress of their therapies for the purpose of attaining health promotion and self-health management.

After Institutional Review Board approval from both hospitals and Tzu Chi University of Science and Technology (Ethical approval code: TCCTIC-1032C018). A face-to-face interview was conducted with a structured questionnaire to collect demographic information, and a five-point Likert scale was used to evaluate the degree of each construct with scores ranging from 1 (strongly disagree) to 5 (strongly agree). The interviewers randomly visited participants, inquiring whether they wanted to participate in this research. Because most of the demographic variables collected were gender, age, educational status, primary caretaker, and chronic disease diagnosed, and the construct questions were adapted from literature and other questionnaires related to the TAM, UTAUT, and PMT. The questions exploring PEOU, PU, subjective norm, behavioral intention, and usage behavior were adapted from Venkatesh and Davis [40,41] and Venkatesh [16], whereas those concerning perceived susceptibility, perceived severity, self-efficacy, response efficacy, and response costs were adapted from Sun, Wang, Guo and Peng [7]. All questions are shown at Table A1 in Appendix A.

A draft of the questionnaire was reviewed and revised in advance by related experts, including two hospital directors, two doctors, and one information engineer. Subsequently, a pretest was conducted with ten participants recruited from the community, whose oral and written feedback was incorporated into the final version of the questionnaire. A total of 450 questionnaires were completed between July and October 2015, each with an interviewer nearby to provide explanation or assistance if necessary.

Based on the requirements of the study and the aforementioned hypotheses, two statistical tools SPSS 18.0 (SPSS Inc., Chicago, IL, USA) and AMOS 17.0 (SPSS Inc., Chicago, IL, USA) were adopted to perform fundamental and overall model analyses, respectively; the results were then verified using structural equation modeling. In all, four statistical analysis methods were used in this study: descriptive statistical analysis, the common method variance (CMV) test, reliability and validity analysis, and structural equation modeling.

## 3. Results

### 3.1. Descriptive Characteristics

There were 223 valid questionnaires available. The effective return rate was 50%. Descriptive statistics revealed that males comprised 39.9% (*n* = 89) and females comprised 60.1% (*n* = 134) of the participants. The majority of the participants were aged more than 70 years (65.5%, *n* = 146), followed by 60–69 years (20.2%, *n* = 45). Furthermore, most of the participants only had a primary school education (42.2%, *n* = 94) or were illiterate (31.8%, *n* = 71). The discussion about the participants’ primary caretakers consisted of one multiple choice question, which revealed that most of the caretakers were the spouse of a participant (56.5%, *n* = 126) or the child of a participant (39.6%, *n* = 113). Finally, the participants identified chronic diseases they had developed: hypertension was the most common (39.5%, *n* = 123) and “other” was the second most common (21.5%, *n* = 67).

### 3.2. Common Method Variance Analysis

The present study used Harman’s single factor test to identify the CMV, which comprised exploratory factor analysis (EFA) and confirmatory factor analysis (CFA).

#### 3.2.1. Exploratory Factor Analysis

First, 29 questions that addressed the ten constructs (i.e., PU, PEOU, subjective norm, behavioral intention, usage behaviors, perceived severity, perceived susceptibility, self-efficacy, response efficacy, and response costs) were used to the conduct the EFA. Nine factors that had an eigenvalue of more than 1 were extracted, resulting in a 92.517% cumulative explained variance. Because the first factor explained 31.993% of the variance (i.e., did not exceed 50%), the study’s data were not seriously distorted by the CMV (Table 1).

#### 3.2.2. Confirmatory Factor Analysis

Next, the 29 questions from the EFA were examined to conduct the single factor CFA. No question was determined to have reached a significant loading (>0.5), which further verified that the study’s data were not seriously distorted by the CMV.

### 3.3. Reliability and Validity

#### 3.3.1. Reliability Analysis

The present study then evaluated the questionnaire’s internal consistency using Cronbach’s α, composite reliability (CR), and the average variance extracted (AVE). First, Cronbach’s α was used to analyze the reliability of the constructs’ Likert scores. According to Guilford [42], internal consistency was confirmed if Cronbach’s α was >0.7. As outlined in Table 2, all of the scales exhibited a Cronbach’s α > 0.7, indicating a favorable internal consistency.

#### 3.3.2. Convergent Validity Analysis

CFA was used to confirm and evaluate the convergent validity of the latent variables with the observed variables, through the CR and AVE of the latent variables [43]. CR measured the combined reliability of all of the observed variables to the latent variables, and AVE measured the explanatory power of the observed variables for the average variance of the latent variables. All of the constructs in the present study had a CR value > 0.7 (Table 2), revealing an internal consistency between the observed variables that were used for the measurement of the latent variables. Therefore, all of the constructs found in the present study exhibited favorable convergent validity.

#### 3.3.3. Discriminant Validity Analysis

The discriminant validity analysis in the present study was adopted from Fornell and Larcker [44], who argued that if the correlation coefficient of two constructs was lower than the square roots of the AVE of each construct, the discriminant validity between these constructs was confirmed. All of the study’s data conformed to this rule, revealing a favorable discriminant validity for the constructs (Table 3).

### 3.4. Structural Equation Modeling

We used the AMOS software (IBM Software, Armonk, NY, USA) for structural model analysis. Overall model fit, which refers to the overall goodness of fit between the model and the data, was conducted according to the suggestion by Hair [43], with the following indicators: (1) absolute fit measures; (2) incremental fit measures; and (3) parsimonious fit measures.

The overall model fit was as follows: χ^2^/df = 1.985, goodness-of-fit index = 0.875, adjusted goodness-of fit index = 0.795, nonnormed fit index = 0.960, comparative fit index = 0.913, incremental fit index = 0.913, and root mean square error of approximation = 0.067. Thus, these indicators all conformed to the standards set by Hair [43] and Jöreskog and Sörbom [45], and revealed a favorable overall model fit in the study’s theoretical model. 

### 3.5. Model and Hypothesis Testing

A path coefficient of 0.460 and *p* < 0.001 revealed that high self-efficacy was correlated with a high PU of the PHR system, which supported H1; a similar path coefficient of 0.465 and *p* < 0.001 suggested that high self-efficacy was also correlated with a high PEOU of the PHR system, which confirmed H2. The correlation between PU and behavioral intention was measured with a path coefficient of 0.172 and *p* < 0.01, which indicated that high PU corresponded to a high level of behavioral intention for the PHR system and substantiated H3. Similarly, the correlation between PEOU and behavioral intention was manifested in a path coefficient of 0.215 and *p* < 0.001, which revealed that high PEOU also corresponded to a high level of behavioral intention for the PHR system and supported H4. Furthermore, subjective norm and behavioral intention were found to be correlated, with a path coefficient of 0.103 and *p* < 0.05, indicating that a high level of subjective norm corresponded to a high level of behavioral intention for the PHR system and validating H5. The correlation between self-efficacy and behavioral intention exhibited a similar path coefficient of 0.202 and *p* < 0.05, signifying that high self-efficacy corresponded to a high level of behavioral intention for the PHR system and confirming H8. Response efficacy and behavioral intention were also correlated, and exhibited a path coefficient of 0.145 and *p* < 0.05, suggesting that high response efficacy corresponded to a high level of behavioral intention for the PHR system and verifying H9. Finally, the correlation between behavioral intention and usage behavior was determined by a path coefficient of 0.653 and *p* < 0.001, which demonstrated that a high level of behavioral intention corresponded to a high level of usage behavior of the PHR system and corroborated H11.

**Figure 2:**
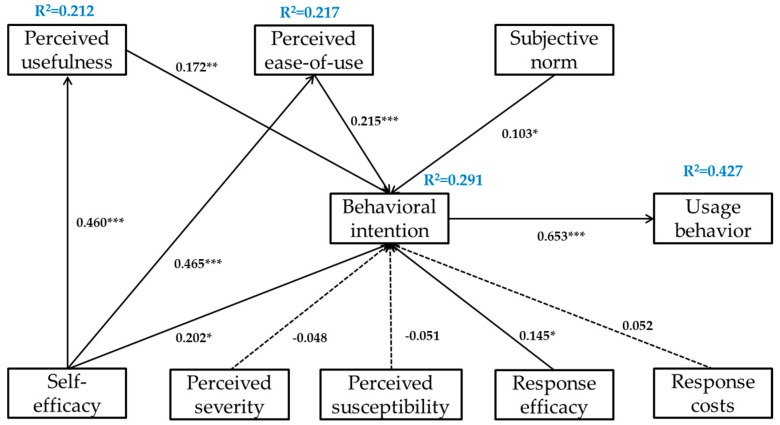
Final proposed model. * *p* < 0.05; ** *p* < 0.01; *** *p* < 0.001.

H6 was rejected following the limited correlation between perceived severity and behavioral intention, defined by a path coefficient of −0.048 and *p* > 0.05. Similarly, perceived susceptibility and behavioral intention demonstrated limited correlation, exhibited by a path coefficient of −0.051 and *p* > 0.05, which invalidated H7. Finally, the correlation between response costs and behavioral intention exhibited a path coefficient of only 0.052 and *p* > 0.05, which nullified H10. All the testing results are summarized in Figure 2.

## 4. Discussion and Findings

Some studies only viewed the PHR as a technical innovation, and have examined it from purely technical perspectives. Other aspects of the PHR have been largely neglected, resulting in blind spots in our understanding of its low popularity. The present study therefore sought to examine factors that affect the use of such a system from technical, medical, and social perspectives by incorporating the TAM and UTAUT models. The results revealed that the comprehensive theory proposed by this study exhibited a favorable overall model fit and explanatory power, offering a suitable basis for future research on the PHR system.

The factors that have a significant impact on the behavioral intention of using the PHR system are perceived ease-of-use, perceived usefulness, self-efficacy, response efficacy, and subjective norm. This finding implies that people in advanced age harbor considerable reservations about the use of modern mobile technology (e.g., smartphones and tablets). The main reason is most of the participants only had a primary school education (42.2%) or were illiterate (31.8%). Additionally, educational seminars presided over by medical staff from the hospital, who can impart medical knowledge and demonstrate the use of PHR, are instrumental in gradually integrating the PHR system into people’s daily health behaviors (e.g., exercise, diet, and taking medication). Finally, social support, such as the support from medical staff, family, and community, is a powerful factor: a positive doctor-patient relationship, support from family and friends, and communal activities suitable for older adults, are all elements of the social context that is indispensable to the well-being of the older population, enabling aging in place.

The positive correlation between perceived severity and behavioral intention in H6 and the positive correlation between perceived susceptibility and behavioral intention in H7 were both found to be invalid. However, further explanation is demanded, because H6 conformed with the results of a study by Gerend and Shepherd [46] and H7 with that of a study by Lajunen and Räsänen [47]. The present author argues that these links can probably be explained by Taiwan’s relatively well-established medical care system that reduces treatment costs to a generally affordable level; this enables people to seek medical attention whenever needed and helps them gain knowledge of the severity of and their susceptibility to diseases. Therefore, the increase in behavioral intention in Taiwan is based on a coping appraisal rather than threat appraisal.

The negative correlation between response costs and behavioral intention in H10 was also found to be invalid, which conformed with the results of a study by Yan et al. [39]. This could potentially be explained by the low cost of the PHR system for community residents. Most of the facilities for the PHR system, including the telehealth system, are provided by the regional hospital free of charge, so the residents are only required to pay for some material costs, such as those for the blood glucose test strips, which are quite affordable. This is probably why response costs would not negatively affect behavioral intention in the study’s results. Further study is recommended to determine if people would be willing to pay for the costs of long-term PHR use at their own expense, or through insurance.

In view of the burgeoning advance of the PHR system in Taiwan, as well as the considerable number of effective samples (*n* = 223) obtained, this study is a valuable reference for the development of local telehealth systems, despite some of the hypotheses being found invalid. Aside from being a reference for health and medical institutions, the study can also be used by academic studies that need to compare domestic data with foreign statistics. Because descriptive statistics have indicated that most PHR users operate the system with the assistance of health advisors or hospital volunteers, this study recommends improved training for people to learn to operate the system on their own, which is expected to enhance their levels of behavioral intention and usage behavior; an increase in the number of educational seminars held is also recommended, because the popularity of the PHR system can improve public health in general.

The scope of the present study is limited to a questionnaire survey of a community covered by the PHR system of a regional hospital in central Taiwan. Thus, future researchers are encouraged to expand the scope by extending the duration of study and recruiting users from other hospitals for a more thorough and conclusive study. It may also be advisable to trace the PHR system’s use in the diagnosis, treatment, and control of certain diseases. Finally, because this study focused on older adults, future studies are recommended to extend the age group to younger adults to determine if age causes any difference in the use of PHR.

## 5. Conclusions

We used PMT and UTAUT to explore the PHR usage behaviors from technical, medical, and social perspectives, with the goal of identifying the decisive factors that could facilitate improved public health and welfare through a widely accepted PHR system. This study recommends that health authorities and medical institutions promote self-efficacy in the use of PHR to improve the levels of behavioral intention and usage behavior among the people. Based on the result, we suggest medical care institutions to promote health management and preventive healthcare concepts to help improve public acceptance of the PHR system. Finally, community centers, medical institutions, and health authorities are urged to work together to enhance public medical knowledge and pool resources for the PHR system, both of which are essential for improving the popularity of the PHR, public quality of life, and the effectiveness of health management.

## Figures and Tables

**Table 1:** Harman’s one-factor test.

Factor	Initial Eigenvalues		
	Total	% of Variance	Cumulative %
1	9.278	31.993	31.993
2	4.013	13.838	45.830
3	2.975	10.259	56.089
4	2.694	9.291	65.380
5	2.404	8.291	73.671
6	1.662	5.730	79.401
7	1.520	5.242	84.643
8	1.191	4.107	88.750
9	1.092	3.767	92.517

**Table 2:** Internal consistency, convergent validity analyses.

Construct	Cronbach’s α	Composite Reliability	Average Variance Extracted
Perceived usefulness	0.992	0.992	0.968
Perceived ease-of-use	0.995	0.995	0.985
Subjective norm	0.937	0.944	0.850
Behavioral intention	0.988	0.997	0.993
Usage behavior	0.979	0.667	0.500
Perceived severity	0.774	0.796	0.574
Perceived susceptibility	0.992	0.992	0.978
Self-efficacy	0.955	0.957	0.883
Response efficacy	0.991	0.992	0.975
Response costs	0.959	0.964	0.900

**Table 3:** Discriminant validity analyses.

Items	Mean	SD	1	2	3	4	5	6	7	8	9	10
1. Perceived usefulness	4.09	0.545	0.984									
2. Perceived ease-of-use	4.07	0.621	0.332 ***	0.992								
3. Subjective norm	3.65	0.899	0.284 ***	0.124	0.922							
4. Perceived severity	3.47	0.850	0.158 *	0.236 ***	0.089	0.758						
5. Perceived susceptibility	2.92	1.11	0.051	0.195 **	0.024	0.599 ***	0.989					
6. Self-efficacy	4.11	0.396	0.392 ***	0.440 ***	0.261 ***	0.243 ***	0.203 **	0.939				
7. Response efficacy	4.10	0.592	0.645 ***	0.376 ***	0.152 *	0.218 **	0.152 *	0.348 ***	0.987			
8. Response costs	2.03	0.656	−0.023	0.019	−0.026	0.093	0.015	−0.053	−0.070	0.949		
9. Behavioral intention	4.15	0.541	0.413 ***	0.393 ***	0.267 ***	0.150 *	0.007	0.432 ***	0.421 ***	0.031	0.426	
10. Usage behavior	3.99	0.656	0.304 ***	0.320 ***	0.329 ***	0.142 *	0.048	0.369 ***	0.248 ***	0.073	0.659 ***	0.309

* *p* < 0.05; ** *p* < 0.01; *** *p* < 0.001.

## Appendix A

**Table A1:** Questionnaire.

Perceived usefulness	Using PHR tool, I can understand my physical condition. Using PHR tool, I can manage my health. Using PHR tool, I can always ask medical staff for advice. Overall, PHR tool is useful for my health.
Perceived ease-of-use	I think the equipment for PHR tool is easy to use. I can easily get familiar with the functions of PHR tool. I think the use of PHR interface is very clear and easy to understand.
Subjective norm	My friends and family use PHR tool. My friends and family thought I should use PHR tool. Because my friends and family use remote personal health records, I use too.
Behavioral intention	I would like to continue to use PHR tool. My willingness to use PHR tool is high.
Usage behavior	I often use PHR tool. I use a lot of PHR tool.
Perceived severity	If too slow to find a serious disease, it will delay the timing of treatment. If I get a serious disease, it will change my whole life. I would be afraid to get serious illness.
Perceived susceptibility	I think I have a high risk to get serious disease. I will worry about my own serious illness. I feel more vulnerable than others.
Self-efficacy	I believe I will use PHR tool. I am confident that I can operate PHR tool. Even no one tell me how to use PHR tool, I still can use it.
Response efficacy	PHR tool can detect serious disease early. PHR tool can monitor blood glucose and other physiological information. PHR tool can let medical staff know my health status.
Response costs	Using PHR tool will take me a lot of time. Using PHR tool will change my lifestyle. Using PHR tool make me feel inconvenient.
